# *catena*-Poly[[(5-carb­oxy-2*H*-1,2,3-triazole-4-carboxyl­ato-κ^2^ *N* ^3^,*O* ^4^)sodium]-di-μ-aqua-κ^4^ *O*:*O*]

**DOI:** 10.1107/S1600536810037384

**Published:** 2010-09-30

**Authors:** Hai-Yan Liu, Li-Xin Liu, Jing-Quan Sha, Lian-Sheng Yu

**Affiliations:** a The Provincial Key Laboratory of Biological Medicine Formulation, School of Pharmacy, Jiamusi University, Jiamusi 154007, People’s Republic of China

## Abstract

In the title coordination polymer, [Na(C_4_H_2_N_3_O_4_)(H_2_O)_2_]_*n*_, the Na^I^ atom is six-coordinated by one O atom and one N atom from a 2*H*-1,2,3-triazole-4-carb­oxy-5-carboxyl­ate ligand and four O atoms from four water mol­ecules, forming a distorted octa­hedal geometry. The Na^I^ atoms are bridged by water mol­ecules into a chain structure along [100]. Inter­molecular N—H⋯O, O—H⋯N and O—H⋯O hydrogen bonds connect the chains. An intra­molecular O—H⋯O hydrogen bond between the carboxyl­ate groups is observed.

## Related literature

For general background to the design and synthesis of metal–organic frameworks (MOFs), see: Chen *et al.* (2009 ▶); Rosi *et al.* (2003 ▶); Su *et al.* (2004 ▶); Xiao *et al.* (2006 ▶). For the use of heterocyclic dicarb­oxy­lic acids in MOFs, see: Gao *et al.* (2006 ▶); Mukherjee *et al.* (2004 ▶); Shi *et al.* (2006 ▶); Sun *et al.* (2005 ▶). For metal complexes with 2*H*-1,2,3-triazole-4,5-dicarb­oxy­lic acid, see: Liu *et al.* (2008 ▶); Yue *et al.* (2008 ▶); Zheng *et al.* (2009 ▶).
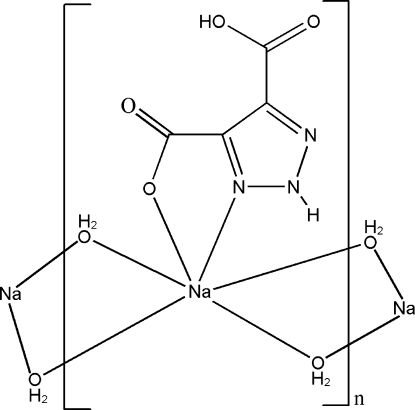


## Experimental

### 

#### Crystal data

[Na(C_4_H_2_N_3_O_4_)(H_2_O)_2_]
*M* *_r_* = 215.11
Monoclinic, 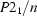

*a* = 6.8706 (9) Å
*b* = 10.6280 (13) Å
*c* = 11.5585 (14) Å
β = 95.647 (1)°
*V* = 839.91 (18) Å^3^
*Z* = 4
Mo *K*α radiation
μ = 0.20 mm^−1^
*T* = 293 K
0.23 × 0.22 × 0.18 mm

#### Data collection

Bruker APEX CCD diffractometer
Absorption correction: multi-scan (*SADABS*; Sheldrick, 1996 ▶) *T*_min_ = 0.955, *T*_max_ = 0.965
4453 measured reflections
1658 independent reflections
1509 reflections with *I* > 2σ(*I*)
*R*_int_ = 0.026

#### Refinement

*R*[*F* ^2^ > 2σ(*F* ^2^)] = 0.041
*wR*(*F* ^2^) = 0.102
*S* = 1.00
1658 reflections
148 parameters
4 restraints
H atoms treated by a mixture of independent and constrained refinement
Δρ_max_ = 0.47 e Å^−3^
Δρ_min_ = −0.59 e Å^−3^

### 

Data collection: *SMART* (Bruker, 2007 ▶); cell refinement: *SAINT* (Bruker, 2007 ▶); data reduction: *SAINT*; program(s) used to solve structure: *SHELXS97* (Sheldrick, 2008 ▶); program(s) used to refine structure: *SHELXL97* (Sheldrick, 2008 ▶); molecular graphics: *SHELXTL* (Sheldrick, 2008 ▶) and *Mercury* (Macrae *et al.*, 2006 ▶); software used to prepare material for publication: *SHELXTL*.

## Supplementary Material

Crystal structure: contains datablocks I, global. DOI: 10.1107/S1600536810037384/hy2351sup1.cif

Structure factors: contains datablocks I. DOI: 10.1107/S1600536810037384/hy2351Isup2.hkl

Additional supplementary materials:  crystallographic information; 3D view; checkCIF report

## Figures and Tables

**Table 1:** Hydrogen-bond geometry (Å, °)

*D*—H⋯*A*	*D*—H	H⋯*A*	*D*⋯*A*	*D*—H⋯*A*
N2—H2⋯O2^i^	0.95 (2)	1.74 (2)	2.652 (2)	158 (2)
O3—H3⋯O1	0.82	1.65	2.468 (2)	177
O5—H5*A*⋯N3^ii^	0.85 (2)	2.15 (2)	2.949 (2)	155 (2)
O5—H5*B*⋯O3^iii^	0.82 (2)	2.13 (2)	2.923 (2)	163 (2)
O6—H6*A*⋯O1^i^	0.84 (2)	2.07 (2)	2.902 (2)	173 (2)
O6—H6*B*⋯O4^iii^	0.80 (2)	2.03 (2)	2.819 (2)	173 (2)

Symmetry codes: (i) 
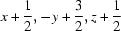
; (ii) 
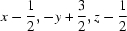
; (iii) 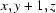.

**Fig. 1.**
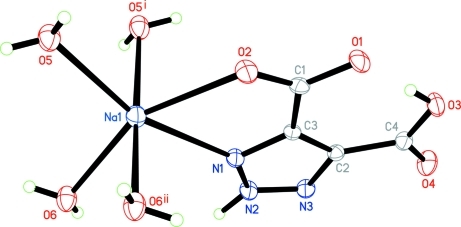
The asymmetric unit of the title compound with symmetry-related atoms to complete the Na coordination. Displacement ellipsoids are drawn at the 30% probability level. [Symmetry codes: (i) -x, 2-y, 2-z; (ii) 1-x, 2-y, 2-z.]

**Fig. 2.**
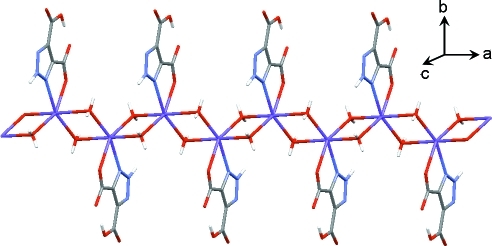
View of the one-dimensional chain in the title compound.

## supplementary crystallographic information

### Comment

Currently, there has been intense research effort on the design and synthesis of metal-organic frameworks (MOFs) owing to their intriguing variety of architectures and their tremendous potential applications in many fields (Chen *et al.*, 2009; Rosi *et al.*, 2003; Su *et al.*, 2004; Xiao *et al.*, 2006). As one kind of well known ligands, heterocyclic dicarboxylic acids have been used to prepare MOFs with multi-dimensional structures because of the hetero atoms may serve as potential coordinating sites (Gao *et al.*, 2006; Mukherjee *et al.*, 2004; Shi *et al.*, 2006; Sun *et al.*, 2005), such as 2*H*-1,2,3-triazole-4,5-dicarboxylic acid (H_2_tda). The three triazole N atoms of H_2_tda can coordinate to various metals (such as Mn, Cd and K), resulting in the formation of intriguing multi-dimensional structures with complicated topologies (Liu *et al.*, 2008; Yue *et al.*, 2008; Zheng *et al.*, 2009).

In the title coordination polymer (Fig. 1), the Na^I^ atom is six-coordinated by one O atom and one N atom from one Htda ligand, and four O atoms from water molecules, with a slightly distorted octahedral geometry. Furthermore, the Na^I^ atoms are bridged by the water molecules, leading to a one-dimensional chain structure, as shown in Fig. 2. Intermolecular N—H···O, O—H···N and O—H···O hydrogen bonds connect the chains (Table 1).

### Experimental

All chemicals were purchased from commercial sources and used without further purification. A mixture of H_2_tda and NaOH in a molar ratio of 1:1 was dissolved in water. Colorless block crystals of the title compound were obtained by slow evaporation of the filtrate over a period of 3 d.

### Refinement

H atoms were located from a difference Fourier map. H3 attached to the carboxyl O3 was refined as riding atom, with O—H = 0.82 Å and *U*_iso_(H) = 1.2*U*_eq_(O). The other H atoms were refined isotropically.

### Crystal data

**Table:**

[Na(C_4_H_2_N_3_O_4_)(H_2_O)_2_]	*F*(000) = 440
*M**_r_* = 215.11	*D*_x_ = 1.701 Mg m^−^^3^*D*_m_ = 1.701 Mg m^−^^3^*D*_m_ measured by not measured
Monoclinic, *P*2_1_/*n*	Mo *K*α radiation, λ = 0.71073 Å
Hall symbol: -P 2yn	Cell parameters from 2984 reflections
*a* = 6.8706 (9) Å	θ = 2.6–28.2°
*b* = 10.6280 (13) Å	µ = 0.20 mm^−^^1^
*c* = 11.5585 (14) Å	*T* = 293 K
β = 95.647 (1)°	Block, colorless
*V* = 839.91 (18) Å^3^	0.23 × 0.22 × 0.18 mm
*Z* = 4	

### Data collection

**Table:**

Bruker APEX CCD diffractometer	1658 independent reflections
Radiation source: fine-focus sealed tube	1509 reflections with *I* > 2σ(*I*)
graphite	*R*_int_ = 0.026
φ and ω scans	θ_max_ = 26.0°, θ_min_ = 2.6°
Absorption correction: multi-scan (*SADABS*; Sheldrick, 1996)	*h* = −8→8
*T*_min_ = 0.955, *T*_max_ = 0.965	*k* = −9→13
4453 measured reflections	*l* = −14→14

### Refinement

**Table:**

Refinement on *F*^2^	Primary atom site location: structure-invariant direct methods
Least-squares matrix: full	Secondary atom site location: difference Fourier map
*R*[*F*^2^ > 2σ(*F*^2^)] = 0.041	Hydrogen site location: inferred from neighbouring sites
*wR*(*F*^2^) = 0.102	H atoms treated by a mixture of independent and constrained refinement
*S* = 1.00	*w* = 1/[σ^2^(*F*_o_^2^) + (0.0608*P*)^2^ + 0.3697*P*] where *P* = (*F*_o_^2^ + 2*F*_c_^2^)/3
1658 reflections	(Δ/σ)_max_ = 0.001
148 parameters	Δρ_max_ = 0.47 e Å^−^^3^
4 restraints	Δρ_min_ = −0.59 e Å^−^^3^

### Fractional atomic coordinates and isotropic or equivalent isotropic displacement parameters (Å^2^)

**Table:**

	*x*	*y*	*z*	*U*_iso_*/*U*_eq_	
H5B	0.091 (4)	1.1695 (18)	0.910 (3)	0.081 (10)*	
H5A	0.028 (4)	1.076 (3)	0.8361 (16)	0.071 (8)*	
H6A	0.503 (3)	1.018 (2)	1.1923 (15)	0.054 (7)*	
H6B	0.433 (4)	1.1154 (18)	1.136 (2)	0.056 (7)*	
Na1	0.25079 (9)	0.92974 (6)	0.99461 (6)	0.0348 (2)	
O1	0.11703 (17)	0.54042 (11)	0.85612 (9)	0.0331 (3)	
O2	0.15004 (18)	0.74783 (11)	0.87493 (10)	0.0347 (3)	
N1	0.34905 (19)	0.72021 (12)	1.09099 (11)	0.0260 (3)	
O6	0.4709 (2)	1.04572 (12)	1.12560 (11)	0.0364 (3)	
O3	0.20462 (18)	0.34772 (11)	0.96846 (10)	0.0348 (3)	
H3	0.1733	0.4126	0.9330	0.042*	
O5	0.0576 (2)	1.09590 (12)	0.90710 (11)	0.0388 (3)	
N3	0.42462 (19)	0.54647 (13)	1.19491 (11)	0.0276 (3)	
O4	0.3474 (2)	0.29745 (11)	1.14248 (11)	0.0420 (3)	
N2	0.4322 (2)	0.67011 (13)	1.18801 (11)	0.0282 (3)	
C2	0.3282 (2)	0.51189 (14)	1.09393 (12)	0.0225 (3)	
C4	0.2933 (2)	0.37598 (15)	1.06997 (14)	0.0279 (3)	
C3	0.2809 (2)	0.62053 (14)	1.02900 (12)	0.0216 (3)	
C1	0.1755 (2)	0.63899 (14)	0.91084 (12)	0.0243 (3)	
H2	0.499 (3)	0.719 (2)	1.2488 (19)	0.045 (6)*	

### Atomic displacement parameters (Å^2^)

**Table:**

	*U*^11^	*U*^22^	*U*^33^	*U*^12^	*U*^13^	*U*^23^
Na1	0.0355 (4)	0.0270 (4)	0.0401 (4)	−0.0001 (3)	−0.0051 (3)	0.0002 (3)
O1	0.0393 (6)	0.0318 (6)	0.0254 (6)	−0.0025 (5)	−0.0109 (5)	−0.0054 (5)
O2	0.0422 (7)	0.0302 (6)	0.0280 (6)	−0.0002 (5)	−0.0147 (5)	0.0058 (5)
N1	0.0289 (7)	0.0246 (7)	0.0226 (6)	0.0010 (5)	−0.0072 (5)	−0.0012 (5)
O6	0.0493 (8)	0.0284 (7)	0.0300 (6)	0.0013 (5)	−0.0030 (5)	−0.0014 (5)
O3	0.0438 (7)	0.0226 (6)	0.0366 (6)	−0.0034 (5)	−0.0025 (5)	−0.0036 (5)
O5	0.0505 (8)	0.0310 (7)	0.0327 (7)	0.0001 (6)	−0.0066 (6)	−0.0020 (5)
N3	0.0309 (7)	0.0281 (7)	0.0226 (6)	0.0028 (5)	−0.0042 (5)	0.0021 (5)
O4	0.0553 (8)	0.0258 (6)	0.0443 (7)	0.0062 (5)	0.0022 (6)	0.0097 (5)
N2	0.0334 (7)	0.0278 (7)	0.0212 (6)	0.0014 (6)	−0.0088 (5)	−0.0025 (5)
C2	0.0216 (7)	0.0241 (8)	0.0215 (7)	0.0018 (5)	−0.0003 (5)	0.0006 (6)
C4	0.0274 (8)	0.0241 (8)	0.0323 (8)	0.0011 (6)	0.0040 (6)	0.0001 (6)
C3	0.0206 (7)	0.0233 (7)	0.0198 (7)	0.0008 (5)	−0.0035 (5)	−0.0007 (5)
C1	0.0221 (7)	0.0292 (8)	0.0204 (7)	0.0004 (6)	−0.0050 (5)	0.0006 (6)

### Geometric parameters (Å, °)

**Table:**

Na1—O5	2.3747 (15)	O3—C4	1.303 (2)
Na1—O6	2.3765 (14)	O3—H3	0.8200
Na1—O2	2.4377 (13)	O5—H5B	0.82 (2)
Na1—O6^i^	2.4855 (15)	O5—H5A	0.85 (2)
Na1—O5^ii^	2.5157 (16)	N3—N2	1.3178 (19)
Na1—N1	2.5510 (14)	N3—C2	1.336 (2)
O1—C1	1.2683 (19)	O4—C4	1.215 (2)
O2—C1	1.2356 (19)	N2—H2	0.95 (2)
N1—N2	1.3196 (18)	C2—C3	1.398 (2)
N1—C3	1.3375 (19)	C2—C4	1.486 (2)
O6—H6A	0.84 (2)	C3—C1	1.4946 (19)
O6—H6B	0.80 (2)		
			
O5—Na1—O6	100.34 (5)	C3—N1—Na1	113.19 (9)
O5—Na1—O2	103.44 (5)	Na1—O6—Na1^i^	100.06 (5)
O6—Na1—O2	154.19 (5)	Na1—O6—H6A	120.0 (17)
O5—Na1—O6^i^	96.45 (5)	Na1^i^—O6—H6A	113.8 (17)
O6—Na1—O6^i^	79.94 (5)	Na1—O6—H6B	112.2 (18)
O2—Na1—O6^i^	87.54 (5)	Na1^i^—O6—H6B	105.3 (19)
O5—Na1—O5^ii^	79.20 (5)	H6A—O6—H6B	105 (2)
O6—Na1—O5^ii^	106.25 (5)	C4—O3—H3	109.5
O2—Na1—O5^ii^	88.03 (5)	Na1—O5—Na1^ii^	100.80 (5)
O6^i^—Na1—O5^ii^	172.90 (5)	Na1—O5—H5B	124 (2)
O5—Na1—N1	161.44 (5)	Na1^ii^—O5—H5B	109 (2)
O6—Na1—N1	92.87 (5)	Na1—O5—H5A	107.1 (19)
O2—Na1—N1	66.65 (4)	Na1^ii^—O5—H5A	105.9 (19)
O6^i^—Na1—N1	98.66 (5)	H5B—O5—H5A	109 (3)
O5^ii^—Na1—N1	84.65 (5)	N2—N3—C2	103.92 (12)
O5—Na1—Na1^i^	100.92 (4)	N3—N2—N1	115.94 (12)
O6—Na1—Na1^i^	41.05 (3)	N3—N2—H2	121.2 (13)
O2—Na1—Na1^i^	123.06 (4)	N1—N2—H2	122.8 (13)
O6^i^—Na1—Na1^i^	38.90 (3)	N3—C2—C3	108.12 (13)
O5^ii^—Na1—Na1^i^	147.18 (5)	N3—C2—C4	119.18 (13)
N1—Na1—Na1^i^	97.61 (4)	C3—C2—C4	132.70 (14)
O5—Na1—Na1^ii^	40.97 (4)	O4—C4—O3	123.18 (15)
O6—Na1—Na1^ii^	107.45 (5)	O4—C4—C2	120.42 (15)
O2—Na1—Na1^ii^	97.09 (4)	O3—C4—C2	116.40 (13)
O6^i^—Na1—Na1^ii^	137.15 (4)	N1—C3—C2	108.42 (12)
O5^ii^—Na1—Na1^ii^	38.23 (3)	N1—C3—C1	119.88 (13)
N1—Na1—Na1^ii^	122.34 (4)	C2—C3—C1	131.70 (13)
Na1^i^—Na1—Na1^ii^	132.86 (4)	O2—C1—O1	125.34 (14)
C1—O2—Na1	121.94 (9)	O2—C1—C3	118.01 (13)
N2—N1—C3	103.59 (12)	O1—C1—C3	116.64 (13)
N2—N1—Na1	142.86 (10)		

Symmetry codes: (i) −*x*+1, −*y*+2, −*z*+2; (ii) −*x*, −*y*+2, −*z*+2.

### Hydrogen-bond geometry (Å, °)

**Table:**

*D*—H···*A*	*D*—H	H···*A*	*D*···*A*	*D*—H···*A*
N2—H2···O2^iii^	0.95 (2)	1.74 (2)	2.652 (2)	158 (2)
O3—H3···O1	0.82	1.65	2.468 (2)	177
O5—H5A···N3^iv^	0.85 (2)	2.15 (2)	2.949 (2)	155 (2)
O5—H5B···O3^v^	0.82 (2)	2.13 (2)	2.923 (2)	163 (2)
O6—H6A···O1^iii^	0.84 (2)	2.07 (2)	2.902 (2)	173 (2)
O6—H6B···O4^v^	0.80 (2)	2.03 (2)	2.819 (2)	173 (2)

Symmetry codes: (iii) *x*+1/2, −*y*+3/2, *z*+1/2; (iv) *x*−1/2, −*y*+3/2, *z*−1/2; (v) *x*, *y*+1, *z*.
